# Evaluation of the Anti-biofilm Activity of Vitamins Against Acinetobacter baumannii and Klebsiella pneumoniae Recovered From Clinical Specimens: An In Vitro and In Silico Analysis

**DOI:** 10.7759/cureus.72679

**Published:** 2024-10-30

**Authors:** Aryan R Ganjo

**Affiliations:** 1 Department of Clinical Analysis, College of Pharmacy, Hawler Medical University, Erbil, IRQ; 2 Department of Medical Analysis, Faculty of Applied Science, Tishk International University, Erbil, IRQ

**Keywords:** acinetobacter baumannii, ascorbic acid, autodock vina, biofilms, cholecalciferol, klebsiella pneumoniae

## Abstract

Introduction: Pathogens that form biofilms reduce the effectiveness of conventional treatments and promote antibiotic resistance. Therefore, this study aimed to investigate the antibiofilm properties of vitamin C (ascorbic acid) and vitamin D (cholecalciferol) experimentally.

Methods: The antibiofilm properties of the studied compounds were evaluated using molecular docking analyses. AutoDock Vina software (The Scripps Research Institute, La Jolla, California) was used to assess the binding affinity of vitamins C and D to the active sites of biofilm-related proteins.

Results: Molecular docking revealed different affinities toward the active sites of the target proteins. The interactions showed promising results, with vitamin D forming both hydrogen bonds and hydrophobic interactions. Compared to vitamin C, vitamin D exhibited the highest binding affinity, with a score of -10.8 kcal/mol.

Conclusion: However, molecular dynamics simulations are needed to further elucidate the dynamic behaviors and stability of these compound-protein complexes. Vitamin D demonstrated good in vitro potential as an anti-biofilm agent and should be considered for use alongside antibiotics in the treatment of bacterial infections.

## Introduction

In recent years, the increased prevalence of *Acinetobacter baumannii *and *Klebsiella pneumoniae *resistant strains has raised serious health concerns. Both pathogens are well-documented as causes of significant infections, such as ventilator-associated pneumonia, liver abscesses, bacteremia, and urinary tract infections, which are associated with considerable morbidity, mortality, and high medical care costs [1]. These bacteria have a remarkable ability to develop resistance and evade the bactericidal activity of nearly all available antibiotics [2]. Another key factor complicating the treatment of infections caused by these pathogens is their ability to form biofilms [3].

Biofilms consist of bacterial communities embedded in extracellular polymeric substances (EPS) that allow them to adhere to inert or living surfaces. Bacteria within biofilms can exchange antimicrobial resistance genes, enhancing their survival in harsh environments and serving as reservoirs for the spread of resistant pathogens [4]. Exposure to antibiotics can increase biofilm tolerance, enabling bacteria to transform into antibiotic-resistant phenotypes.

Vitamin C, a frequently prescribed nutritional supplement, is known for its antioxidant properties. It is water-soluble and synthesized from glucuronic acid and ketolactone, with two ionizable hydroxyl groups, and has few side effects [5]. Vitamin D (calcitriol, or 1,25-dihydroxyvitamin D3) is fat-soluble and plays a critical role in maintaining calcium balance and promoting bone mineralization. In addition to its well-known effects on calcium metabolism, vitamin D also acts as a powerful antioxidant with non-calcemic effects [6]. This vitamin offers a wide range of health benefits and influences several metabolic processes in the body [7].

The molecular docking approach provides valuable insights into chemical interactions at the molecular level, helping to understand how compounds bind to specific targets [8]. The binding affinity of vitamins C and D to biofilm-related proteins suggests their potential effectiveness in disrupting biofilm formation [9].

This study aimed to investigate the biofilm prevalence of *A. baumannii *and *K. pneumoniae *alongside their resistance profiles. It also evaluated the effects of vitamins C and D on biofilm development in vitro, as well as the binding interactions of these vitamins with biofilm-producing protein active sites using molecular docking.

## Materials and methods

Bacterial strains and antimicrobial susceptibility testing (AST)

In the current study, a total of 50 non-duplicative strains of *A. baumannii* and 50 strains of *K. pneumoniae *were isolated. These isolates were obtained from sputum, wound, blood, CSF, and urine specimens collected from patients in the intensive care unit (ICU) as well as from patients not admitted to the ICU. The clinical laboratory received the samples and used standard testing procedures to identify the isolates and determine their antibiotic susceptibility. Each specimen was subjected to conventional biochemical tests before being run through the Vitek 2 system (BioMérieux, France) for additional confirmation.

Biofilm production by microtiter plate technique (MTP)

Pure colonial strains were cultured on Columbia agar supplemented with 5% sheep blood and incubated overnight at 37 °C. Luria Bertani (LB) broth, prepared with a diluted colony, was incubated at 37 °C for 24 h. A 0.5 McFarland standard was used to dilute the solution 100-fold in fresh LB medium. A sterile 96-well microtiter plate was then loaded with 200 µL of the diluted solution. Fresh LB broth served as the negative quality control. Plates were incubated at 37 °C for 48 h. After incubation, the broth was drained by flipping the plate over. Water was added to each well, and the plate was gently shaken. This washing procedure was repeated twice. Subsequently, 250 µL of a 0.1% crystal violet solution was added to each well. After incubating the microtiter plate at room temperature for 10 minutes, the plate was washed three times by immersing it in water and shaking it. The plate was then left to dry completely, positioned upside down for a few hours. The modified MTP method was used to quantify biofilm formation. To dissolve the crystal violet, 250 µL of 95% ethanol was added to each well, and the absorbance was measured at a wavelength of 590 nm. Sterile BHI was used as the negative control. Biofilm-producing *P. aeruginosa *ATCC 27853 served as the positive control. Ultimately, the optical density (OD) of each well was measured at 490 nm. The OD cutoff was defined as three times the standard deviation (SD) above the mean OD of the negative control. Biofilm formation was categorized into three levels: weak, moderate, and strong biofilm formers [9].

Evaluation of vitamins' effect on dissolving bacterial biofilms

The microtiter plate method was used to evaluate the impact of vitamins D and C on *A. baumannii *and *K. pneumoniae *biofilm-producing isolates, following the protocol established by [5]. Freshly prepared broths of *A. baumannii* and *K. pneumoniae *in peptone water were incubated for two hours. LB broth was supplemented with L-ascorbic acid and cholecalciferol, adjusting the final ascorbic acid concentration to 20 mg/mL in each of three different batches. These concentrations were chosen according to the findings of Abdelraheem et al. [10]. For each strain, the positive control consisted of bacterial biofilms treated with sterile LB broth (not exposed to vitamin C). The 96-well microtiter plates were inoculated with 190 µL of bacterial suspension in LB broth at a concentration of 0.5 McFarland. All wells, except the positive and negative controls, received ascorbic acid at a concentration below the minimum inhibitory concentration (MIC). Plates were incubated at 37 °C for 24 h. After incubation, the contents of the wells were carefully emptied. The wells were washed with phosphate-buffered saline (PBS) to remove any remaining bacteria. Bacterial biofilms that had been air- and heat-fixed at 60 °C for one hour were stained with 0.1% crystal violet. Excess stain was removed by washing the wells with water. After 15 minutes of incubation with 95% ethanol, the optical densities (ODs) of the stained bacteria were measured using an ELISA reader at 590 nm. The OD values reflected the extent of biofilm formation. Each experiment was conducted in triplicate, and the results were averaged for analysis.

Computational methods

Preparation of Biofilm Producer Protein and Vitamins (D and C)

The X-ray crystal structures of the biofilm producer proteins of *A. baumannii *(PDB IDs: 3P2H, 3DX8) and *K. pneumoniae *(PDB IDs: 4LFU, 5KEC) were used, respectively. These structures were retrieved from the Research Collaboratory for Structural Bioinformatics (RCSB) Protein Data Bank (PDB) (http://www.rcsb.org) [11]. The 2D structures of vitamins D and C were obtained from PubChem (https://pubchem.ncbi.nlm.nih.gov/) [12]. Discovery Studio 4.1 (http://accerys.com) and Molecular Graphics Laboratory (MGL) Tools 1.5.6 (http://mgltools.scripps.edu) were employed for structural analysis. The X-ray crystal structures of the biofilm-producing proteins from *A. baumannii* and *K. pneumoniae *were used to prepare the protein and vitamin files, which were saved in pdbqt format as required for AutoDock Vina (The Scripps Research Institute, La Jolla, California) [13].

AutoDock Vina

AutoDock Vina was executed on a Windows 8.1 operating system with 4 CPUs and 12 GB of RAM. The configuration file provided coordinates for AutoDock Vina, including the biofilm-producing proteins (receptors), vitamins (ligands), and the dimensions of the active site. A grid was defined with a line cube of 16 Å and a grid point spacing of 1.0 Å. The center grid boxes (X, Y, Z) were set at (17.869, 63.131, 10.143) and (26.503, 28.402, 1.86) for quorum sensing proteins. The quorum sensing proteins included abaI (PDB ID: 3P2H) and OmpA (PDB ID: 3DX8) genes in *A. baumannii* and SdiA (PDB ID: 4LFU), acting as a LuxR receptor, and mrkA (PDB ID: 5KEC) in *K. pneumoniae *[14].

Statistical Analysis

GraphPad Prism software (San Diego, California) was used for all statistical tests. The chi-square test and Fisher’s exact test were employed for comparisons. A p-value less than 0.05 was considered statistically significant.

## Results

During the study, 50 non-repetitive *A. baumannii *and 50 *K. pneumoniae *strains were isolated from patient samples. The majority of *A. baumannii *and *K. pneumoniae *strains were obtained from males (n = 29 (58%) and 32 (64%), respectively). With respect to age, *A. baumannii *strains were more common among patients aged 41-60 (n = 18 (36%)), and *K. pneumoniae *strains were more prevalent among those aged 21-40 (n = 18 (36%)). Additionally, a significantly higher percentage of *A. baumannii *and *K. pneumoniae *strains were recorded from urban settings (Table 1).

**Table 1:** Characteristics of patients from whom A. baumannii and K. pneumoniae were isolated Chi-square test was performed.

Characteristic		*A. baumannii,* n(%)	*K. pneumoniae, *n(%)	P-value
Gender	Male	29(58%)	32(64%)	0.5385
	Female	21(42%)	18(36%)	
Age	≤20	8(16%)	3(6%)	0.3024
	21-40	17(34%)	18(36%)	
	41-60	18(36%)	17(34%)	
	≥61	7(14%)	12(24%)	
Geographical setting	Urban	41(82%)	49(98%)	0.0077
	Rural	9(18%)	1(2%)	
Sampling location	ICU patient	30(6%)	44(88%)	0.0014
	Non-ICU patient	20(4%)	6(12%)	
Gender, ICU patient	ICU patient - Male	22(44%)	29(58%)	0.4981
	ICU patient - Female	8(16%)	15(30%)	
Gender, non-ICU patient	non-ICU patient - Male	7(14%)	3(6%)	0.5077
	non-ICU patient - Female	13(26%)	3(6%)	
Antibiotic usage (last 2 weeks)	Yes	35(70%)	42(84%)	0.0962
	No	15(30%)	8(16%)	

The isolates were resistant to various antibiotics, including ciprofloxacin (28/30; 93.3%) in biofilm producers and (20/20; 100%) in non-biofilm producers. Among *A. baumannii *isolates, 100% (50/50) exhibited resistance to piperacillin, piperacillin/tazobactam, imipenem, meropenem, gentamicin, levofloxacin, and trimethoprim/sulfamethoxazole. Resistance to tigecycline was observed in 10% (3/30) of biofilm producers and 15% (3/20) of non-biofilm producers (Table 2).

**Table 2:** Antibiotic resistance between biofilm-producing and non-biofilm-producing A. baumannii Fisher’s exact test was performed.

Antibiotics	Resistant biofilm producer, n=30	Resistant non-biofilm producer, n=20	P value
Piperacillin	30(100%)	20(100%)	0.9991
Piperacillin/tazobactam	30(100%)	20(100%)	
Imipenem	30(100%)	20(100%)	
Meropenem	30(100%)	20(100%)	
Gentamicin	30(100%)	20(100%)	
Netilmicin	23(76.7%)	18(90%)	
Tobramycin	20(66.6%)	19(95%)	
Ciprofloxacin	28(93.3%)	20(100%)	
Levofloxacin	30(100%)	20(100%)	
Tigecycline	3(10%)	3(15%)	
Tri/sulfamethoxazole	30(100%)	20(100%)	
Colistin	0	0	

The vast majority of *K. pneumoniae* strains tested were resistant to multiple classes of antibiotics. Resistance to fluoroquinolones and third- and fourth-generation cephalosporins was particularly notable in *K. pneumoniae *strains. Among biofilm producers, *K. pneumoniae *exhibited good sensitivity to tigecycline in 25.8% of cases (8/31), while among non-biofilm producers, sensitivity was observed in only 5.3% (1/19) (Table 3).

**Table 3:** Antibiotic resistance between biofilm-producing and non-biofilm-producing K. pneumoniae Fisher’s exact test was performed.

Antibiotics	Resistant biofilm producer, n=31	Resistant non-biofilm producer, n=19	P value
Ampicillin	31(100%)	19(100%)	0.9739
Amoxicillin/clavulanic acid	28(90.3%)	17	
Piperacillin/tazobactam	18(58%)	7(36.8%)	
Amikacin	15(48.3%)	4(21.05%)	
Gentamicin	14(45.1%)	5(26.3%)	
Meropenem	10(32.3%)	4(21%)	
Imipenem	9(29%)	4(21%)	
Ertapenem	11(35.4%)	3(15.8%)	
Ciprofloxacin	15(48.4%)	4(21%)	
Levofloxacin	13(41.9%)	6(31.6%)	
Tigecycline	8(25.8%)	1(5.3%)	
Trimethoprim/sulfamethoxazole	22(71%)	9(47.4%)	
Ceftazidime	25(80.6%)	11(57.9%)	
Cefepime	27(87.1%)	14(73.7%)	
Ceftriaxone	27(87.1%)	12(63.2%)	
Cefuroxime	29(93.5%)	11(57.9%)	
Cefixime	29(93.5%)	12(63.2%)	

In the present study, 50 *A. baumannii* isolates were recovered from the following clinical specimens: sputum (n = 35; 70%), wound (n = 6; 12%), blood (n = 7; 14%), and CSF (n = 2; 4%). No isolates were recovered from urine. Additionally, 60% of all isolates were biofilm producers (Table 4).

**Table 4:** Biofilm production in A. baumannii from different clinical specimens Fisher’s exact test was performed.

Clinical specimens	A. baumannii, n(%)				Total	P-value
	Non-biofilm producer	Weak biofilm producer	Moderate biofilm producer	Strong biofilm producer		
Sputum	15(30%)	7(14%)	5(10%)	8(16%)	35(70%)	0.5287
Wound	2(4%)	1(2%)	1(2%)	2(4%)	6(12%)	
Blood	2(4%)	2(4%)	3(6%)	0	7(14%)	
CSF	1(2%)	1(2%)	0	0	2(4%)	
Urine	0	0	0	0	0	
Total	20(40%)	11(22%)	9(18%)	10(20%)	50(100%)	

While 50 *K. pneumoniae* isolates were obtained from clinical specimens, including sputum (n = 34; 68%), wound (n = 5; 10%), blood (n = 4; 8%), cerebrospinal fluid (n = 3; 6%), and urine (n = 4; 8%), 62% of the *K. pneumoniae* isolates were biofilm producers (Table 5).

**Table 5:** Biofilm production in K. pneumoniae from different clinical specimens

Clinical Specimens	K. pneumoniae, n(%)				Total	P value
	Non-biofilm producer	Weak biofilm producer	Moderate biofilm producer	Strong biofilm producer		
Sputum	14(28%)	10(20%)	7(14%)	3(6%)	34(68%)	0.5000
Wound	0	2(4%)	1(2%)	2(4%)	5(10%)	
Blood	2(4%)	1(2%)	1(2%)	0	4(8%)	
CSF	1(2%)	2(4%)	0	0	3(6%)	
Urine	2(4%)	1(2%)	1(2%)	0	4(8%)	
Total	19(38%)	16(32%)	10(20%)	5(10%)	50(100%)	

The quantification of biofilm using the conventional MTP method divided *A. baumannii* and *K. pneumoniae *isolates into three categories: weak, moderate, and strong adherent. According to the current findings, 11 (36.7%) and 16 (51.6%) of the isolates were classified as weak producers, 9 (30%) and 10 (32.3%) as moderate producers, and 10 (33.3%) and 5 (16.1%) as strong biofilm producers, respectively. Biofilm inhibition of the bacterial strains by Vitamin C was lower compared to Vitamin D, with inhibition rates of 7 (23.3%) and 6 (19.4%) for *A. baumannii *and *K. pneumoniae*, respectively (Table 6).

**Table 6:** Effects of vitamins D and C on biofilm formation in A. baumannii and K. pneumoniae, n(%). A chi-square test was performed. P-value = 0.2685.

Biofilm type	A. baumannii, n(%)			K. pneumoniae, n(%)		
	n(%)	Vitamin C, n(%)	Vitamin D, n(%)	n(%)	Vitamin C, n(%)	Vitamin D, n(%)
Weak biofilm producer	11(36.7%)	5(16.7%)	6(20%)	16(51.6%)	2(6.5%)	5(16.2%)
Moderate biofilm producer	9(30%)	0	1(3.3%)	10(32.3%)	1(3.2%)	0
Strong biofilm producer	10(33.3%)	0	0	5(16.1%)	2(6.5%)	1(3.2%)
Total	30(100%)	5(16.7%)	7(23.3%)	31	5(16.2%)	6(19.4%)

**Table 7:** Average docking scores (kcal/mol) for vitamins D and C docked to biofilm producer proteins * Average of six runs performed for each compound.

Bacteria	Protein PDB-ID	Docked compound	Docking score* (kcal/mol)
K. pneumoniae	4LFU (SdiA)	Vitamin D	-10.8
	4LFU (SdiA)	Vitamin C	-5.3
	5KEC (MrkA)	Vitamin D	-7.0
	5KEC (MrkA)	Vitamin C	-5.5
A. baumannii	3P2H (abaI)	Vitamin D	-6.9
	3P2H (abaI)	Vitamin C	-5.1
	3DX8 (OmpA)	Vitamin D	-9.3
	3DX8 (OmpA)	Vitamin C	-4.9

**Figure 1:**
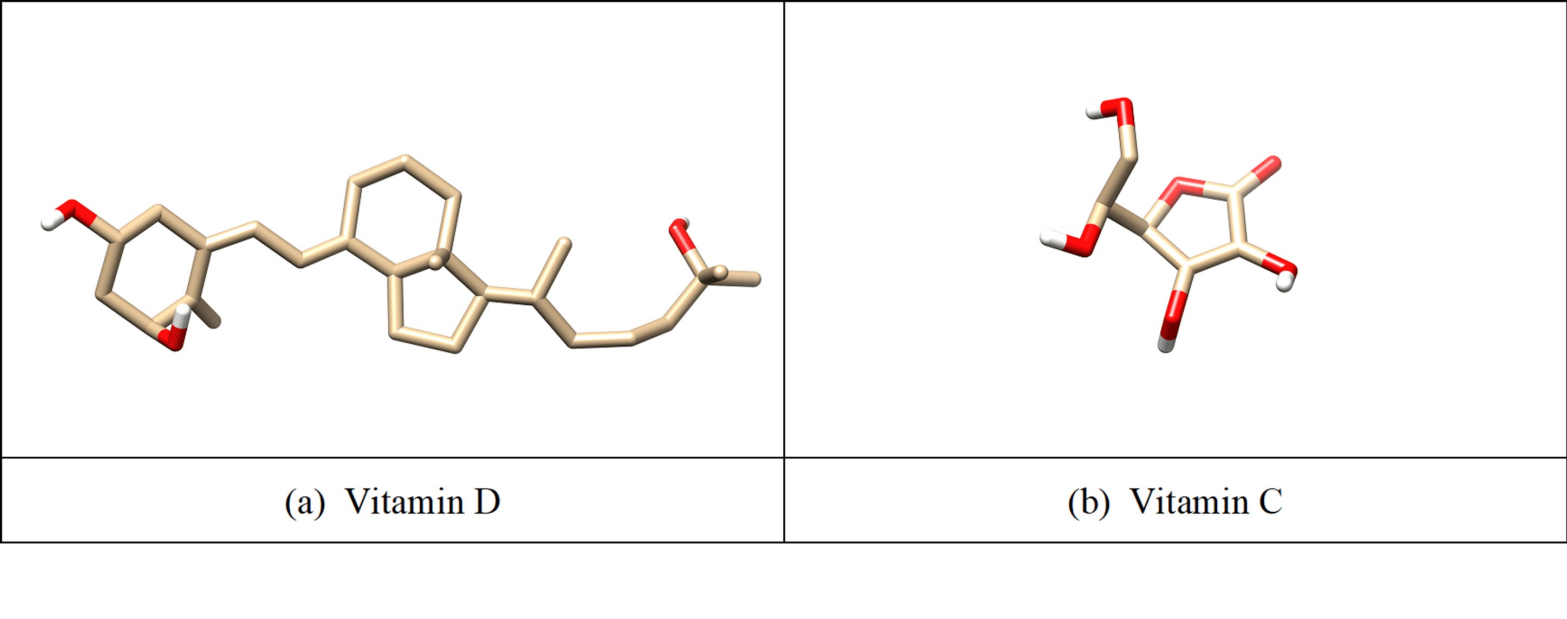
The 3D structure of vitamin D (a) and vitamin C (b) AutoDock Vina software package (version 1.1.2, The Scripps Research Institute, La Jolla, California).

The in-silico study supported the in vitro experiments. A higher affinity was observed when vitamin D (Figure 1) docked into the protein binding sites of *K. pneumoniae *(PDB ID: 4LFU (SdiA); 5KEC (MrkA)) and *A. baumannii *(PDB ID: 3P2H (abaI); 3DX8 (OmpA)), revealing that vitamin D interacts more effectively than vitamin C (Figure 1) with the biofilm-producing proteins of *K. pneumoniae *and *A. baumannii*.

**Figure 2:**
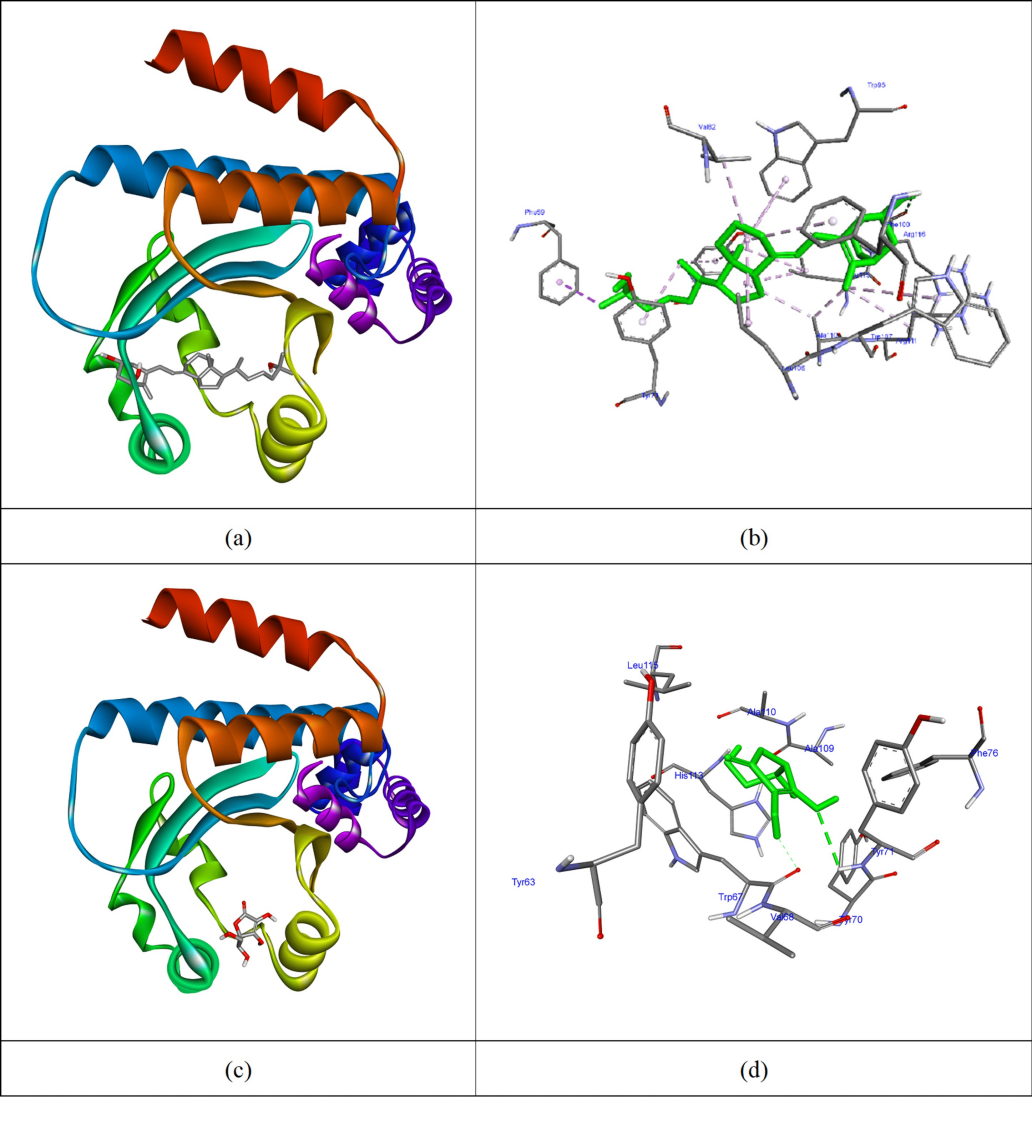
Vitamins D and C docked to 4LFU protein (a) and (c) The molecular surface of 4LFU protein and vitamins D and C in the active site of the protein; (b) and (d) Interacted residues of 4LFU protein with vitamins D and C (AutoDock Vina software package, version 1.1.2, The Scripps Research Institute, La Jolla, California).

Docking scores indicate that vitamin D reduces biofilm production, weakening biofilm activity for *K. pneumoniae*. The effects of vitamins D and C on *A. baumannii *include converting biofilm producers (weak, moderate, or strong) to non-producers and eliminating biofilm production (Table 7). The highest affinity (-10.8 kcal/mol) was observed when vitamin D docked with the 4LFU protein (Table 7, Figure 2). It formed hydrogen bonds with Arg116 and hydrophobic interactions with PHE59, TYR63, TYR71, VAL82, TRP95, PHE100, LEU106, ALA110, ARG111, TRP107, and LEU115. In comparison, vitamin C formed hydrogen bonds with TRP67 and TYR71, yielding a docking score of -5.3 kcal/mol, demonstrating that vitamin D interacts more effectively with the biofilm-producing protein. The active sites of proteins involved in biofilm production play a crucial role in the development and maintenance of bacterial biofilms. Understanding these sites is essential for devising effective strategies to inhibit biofilm formation, particularly for pathogens like *A. baumannii* and *K. pneumoniae*.

**Figure 3:**
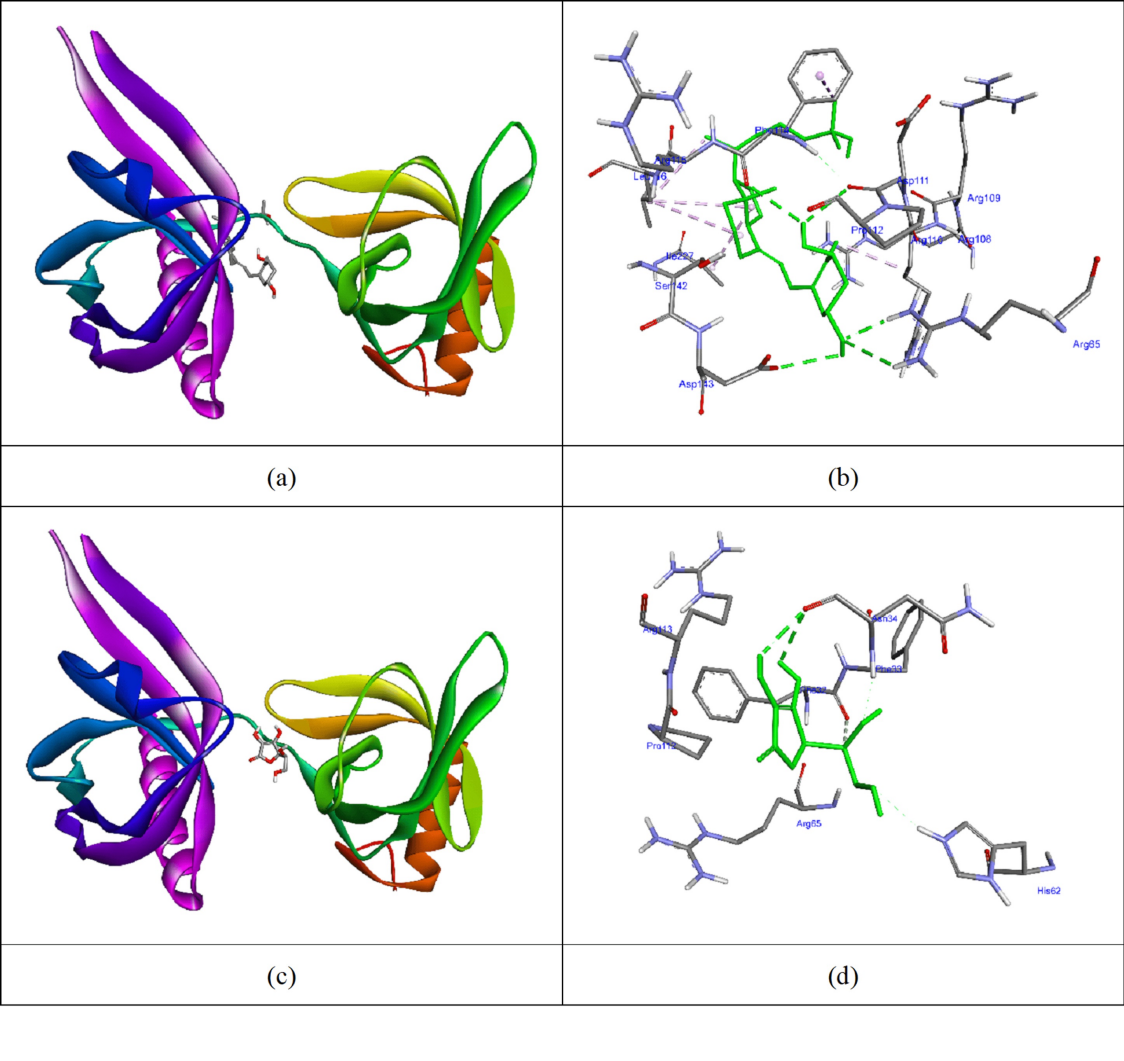
Vitamins D and C docked to 5KEC protein (a) and (c) The molecular surface of 5KEC protein and vitamins D and C in the active site of the protein; (b) and (d) Interacted residues of 5KEC protein with vitamins D and C. (AutoDock Vina software package, version 1.1.2, The Scripps Research Institute, La Jolla, California).

Vitamins D and C docked with the 5KEC protein (Figure 3). Vitamin D exhibited a docking score of -7.0 kcal/mol, forming hydrogen bonds with ARG65, ASP111, PHE114, and ASP143, along with hydrophobic interactions with ARG110, PHE114, LEU116, and ILE227. In comparison, vitamin C formed hydrogen bonds with GLN207, ASN34, and PHE32, yielding a docking score of -5.5 kcal/mol.

**Figure 4:**
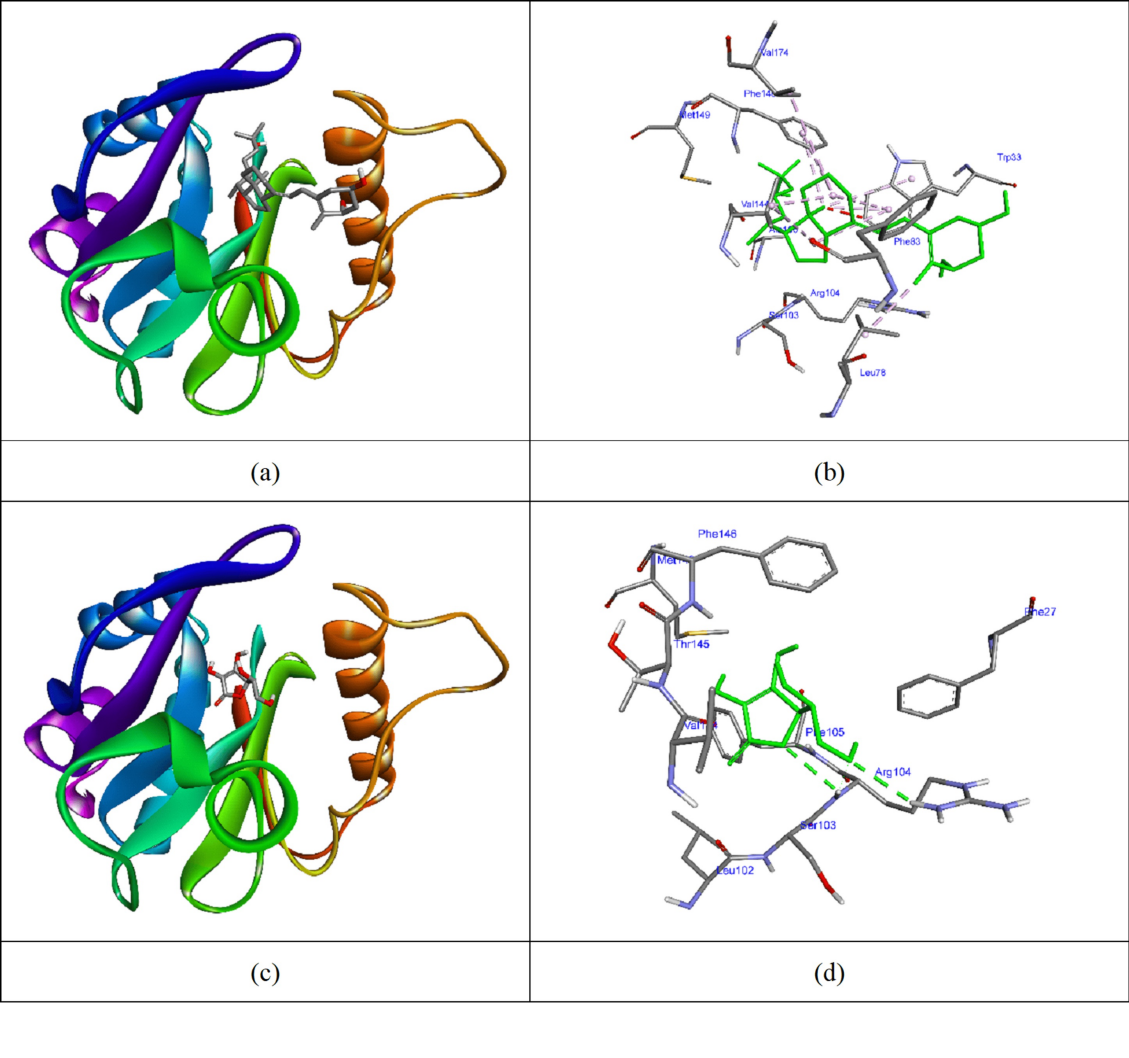
Vitamins D and C docked to 3P2H protein (a) and (c) The molecular surface of 3P2H protein and vitamin D and C in the active site of the protein; (b) and (d) Interacted residues of 3P2H protein with vitamins D and C (AutoDock Vina software package, version 1.1.2, The Scripps Research Institute, La Jolla, California).

Vitamins D and C docked with the 3P2H protein (Figure 4). Vitamin D achieved a docking score of -6.9 kcal/mol, forming hydrophobic interactions with TRP33, LEU78, VAL144, PHE146, and VAL174. In comparison, vitamin C formed hydrogen bonds with SER103, ARG104, and PHE105, yielding a docking score of -5.1 kcal/mol.

**Figure 5:**
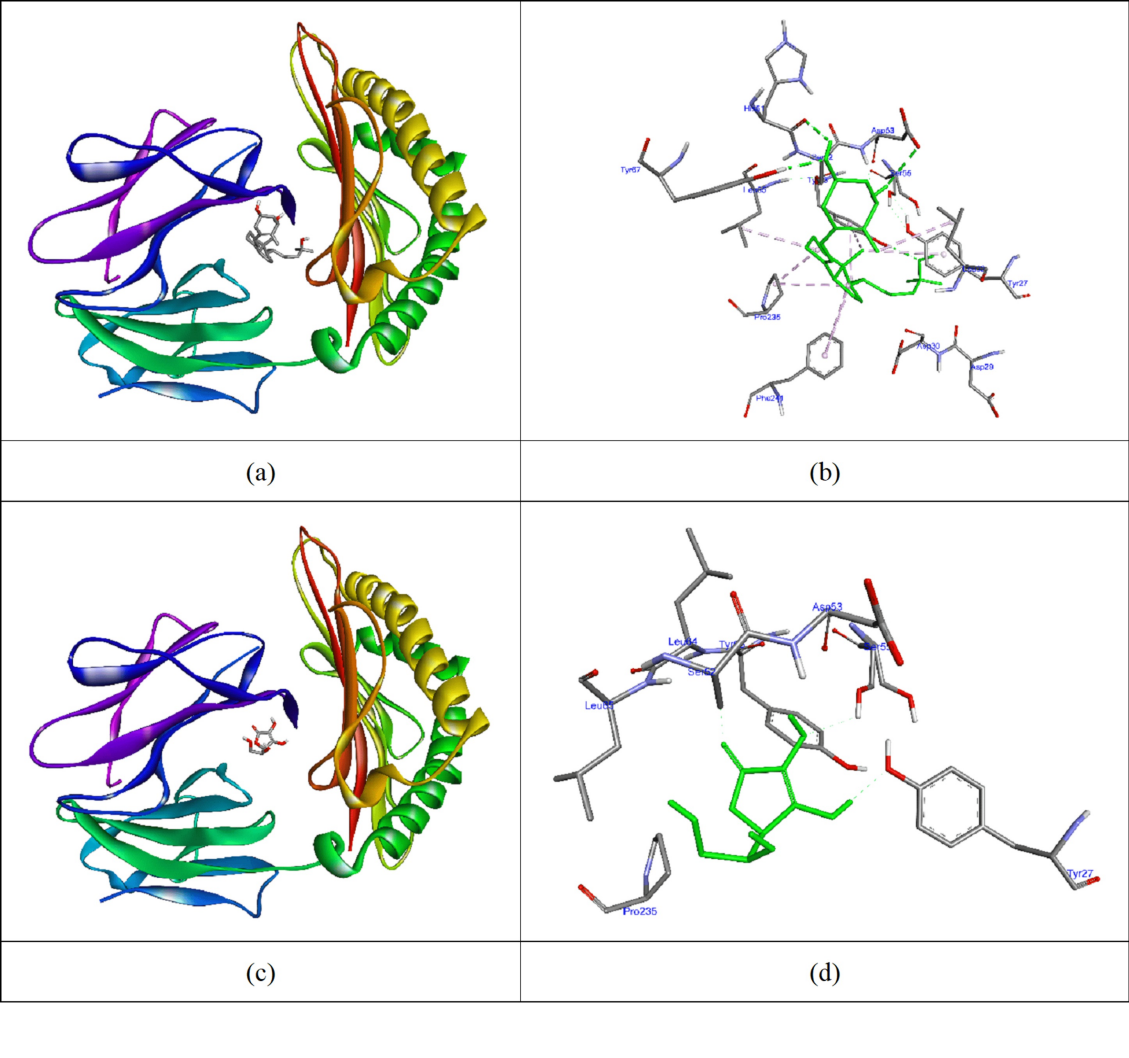
Vitamins D and C docked to 3DX8 protein (a) and (c) The molecular surface of 3DX8 protein and vitamins D and C in the active site of the protein; (b) and (d) Interacted residues of 3DX8 protein with vitamins D and C (AutoDock Vina software package, version 1.1.2, The Scripps Research Institute, La Jolla, California).

Vitamins D and C docked with the 3DX8 protein (Figure 5), which achieved a docking score of -9.3 kcal/mol, forming hydrogen bonds with HIS51, ASP53, TYR63, and TYR67, along with hydrophobic interactions with TYR27, LEU32, TYR63, LEU65, PRO235, and PHE241. In comparison, vitamin C formed hydrogen bonds with TYR27, SER52, SER55, ASP53, and LEU65, yielding a docking score of -4.9 kcal/mol.

## Discussion

These days, Gram-negative bacteria, such as *A. baumannii *and *K. pneumoniae*, are the most common causes of health-related illnesses across various age and gender groups in many countries [15], including ventilator-associated pneumonia, which is prevalent in patients receiving mechanical ventilation [2]. Both bacteria can enter the bloodstream, causing bacteremia, a severe and potentially life-threatening condition. They are also implicated in urinary tract infections (UTIs), especially in hospitalized patients, and are notorious for causing infections in individuals with weakened immune systems, such as those undergoing chemotherapy or living with chronic diseases [1].

Although several epidemiological models exist for studying biofilms in clinical isolates, *A. baumannii* and *K. pneumoniae *were chosen for the present investigation due to the marked rise in carbapenem resistance rates among clinical isolates in the hospital. The majority of these isolates were obtained from male patients, corroborating findings by Qader et al. [16], who reported that male patients are more likely to develop infections than female patients. Negative lifestyle habits, such as smoking and alcohol consumption, were linked to higher infection rates. Bacterial infections were most commonly observed in patients aged 21 to 60 (Table 1). This finding aligns with previous research showing that most bacterial isolates came from patients aged 40 to 65. Another study found that patients over the age of 65 were more likely to have bacterial infections [17].

*K. pneumoniae* and *A. baumannii *isolates predominated in the intensive care unit (ICU), emphasizing that these bacteria commonly colonize the human oropharyngeal and gastrointestinal mucosal surfaces. Consequently, *K. pneumoniae *and *A. baumannii *are responsible for most hospital-acquired pneumonia cases worldwide. Despite colistin being the most potent antibiotic, all strains in this study were sensitive to it (Table 2). The bacteria, however, exhibited significant resistance to antibiotics commonly used to treat Gram-negative infections, but no colistin resistance was detected. This is particularly important, as colistin remains one of the few viable treatment options for drug-resistant Gram-negative bacterial infections [18].

It has been demonstrated that certain medications are not useful against infections. Resistance in these bacteria can result from various factors, including low outer membrane permeability, the presence of β-lactamase or AmpC enzymes, and the activity of multiple drug efflux pumps [19]. Similar results were reported by Nirwati et al. [20], who found that 148 isolates were biofilm producers, with 45 (26.95%) classified as strong biofilm producers, 48 (28.74%) as moderate, and 50 (29.94%) as weak biofilm producers. Another study from Iraq reported that 82.5% of isolates were strong biofilm producers, while 5% were classified as weak producers [21]. Several factors, including the physical properties of the pathogen, the surface to which the biofilm adheres, and environmental conditions such as temperature and pH, can influence biofilm formation potential [22].

In this study, 35 (70%) of *A. baumannii *and 34 (68%) of *K. pneumoniae *isolates from sputum samples were biofilm producers. This finding aligns with previous research indicating that 76.9% (173/225) of sputum samples contained *A. baumannii *isolates with biofilm-forming potential [23]. Another study found that vitamin C inhibited the growth of pathogens such as *E. coli*, *K. pneumoniae*, and *S. aureus *[24]. Furthermore, vitamin C, at lower concentrations, effectively inhibited *P. aeruginosa *biofilm formation. With the search for novel, potent antibacterials at a standstill, there is growing concern about a resurgence of “pre-antibiotic era” diseases [10]. Researchers are urgently working to develop regulations and norms to govern antibiotic use, particularly in developing countries, while also exploring alternative therapeutic approaches to combat antimicrobial resistance [25].

The findings of this study suggest that vitamin D3 may serve as a prophylactic measure against microbial infections. Research shows that vitamin D directly inhibits the growth of *Streptococcus *spp. [26]. Additionally, vitamin D regulates the production of endogenous antimicrobial peptides and defensins, which exhibit broad-spectrum antibacterial activity against various pathogens [27]. However, comparisons with other studies were limited, as research on the anti-biofilm activities of vitamin D against *A. baumannii* and *K. pneumoniae *remains scarce.

The docking protocol was successfully implemented, and vitamins D and C were successfully docked into the active sites of biofilm-producing proteins. Ten runs were performed for each vitamin, and the average docking scores (kcal/mol) were calculated (Table 7). A study highlights that vitamins C and D interact with specific active sites of biofilm proteins through molecular docking. These interactions, characterized by hydrogen bonds and hydrophobic contacts, suggest that the complexes may play a role in inhibiting the function of these proteins, which are crucial for biofilm formation [11].

The interactions between the vitamins and the active sites involve hydrogen bonds and hydrophobic contacts. For instance, vitamin D formed multiple hydrogen bonds and hydrophobic interactions with various amino acids in the protein structure. These interactions are significant because they stabilize the binding of the vitamin to the protein, potentially inhibiting its activity and, consequently, biofilm production. Proper orientation within the active site was essential for these interactions, allowing the formation of stable protein-ligand complexes.

Vitamin C was found to exhibit significant anti-biofilm, anti-virulence, and bactericidal activities against hypervirulent *K. pneumoniae*, with a dose-dependent bactericidal effect, as reported by Xu et al. [28]. Additionally, vitamins have the potential to boost immunity, prevent the spread of pathogens, and reduce the disease progression to advanced stages [29].

Future recommendations

Further research is needed to emphasize molecular dynamics studies to better understand the interactions and stability of vitamin compounds with biofilm proteins. This could lead to more effective treatment strategies against infections caused by *A. baumannii *and *K. pneumoniae*. In vivo studies are also necessary to evaluate the effectiveness of vitamins C and D in reducing biofilm formation in animal models, helping to assess the applicability of the findings from in vitro studies.

Limitations of the study

This study was conducted in vitro, which may not accurately represent the environment in which biofilms form during human infections. Although molecular docking was employed to assess the interactions between vitamins and biofilm proteins, the study lacks molecular dynamics simulations that could provide insights into the dynamic behavior and stability of these complexes over time. Additional research is required to elucidate the precise chemical processes underlying the antibiofilm properties of these vitamins.

## Conclusions

The study concluded that a significant percentage of the isolated strains were biofilm producers, with only 40% of *A. baumannii *and 38% of *K. pneumoniae *showing non-biofilm-forming capabilities. Vitamins, particularly vitamin D, demonstrated promising in vitro anti-biofilm activity against the tested bacteria. Molecular docking analysis revealed that vitamin D had a higher binding affinity to biofilm proteins compared to vitamin C, suggesting its potential as a therapeutic agent for managing biofilm-related infections. The findings highlight the potential role of vitamins as safe and effective antimicrobial and antibiofilm agents, contributing to the fight against antimicrobial-resistant microorganisms. Both the in vitro and molecular docking analyses identified vitamin D as the most effective compound for eliminating biofilm production, resulting in a substantial reduction in biofilm activity in *A. baumannii *and *K. pneumoniae*.
